# Some Observations on the Salivary Digestion of Starch by Infants*Read before the College of Physicians of Philadelphia, June 6, 1883.

**Published:** 1883-08

**Authors:** J. M. Keating

**Affiliations:** Visiting Obstetrician to the Philadelphia Hospital, and Lecturer on Diseases of Women and Children


### Some Observations on the Salivary Digestion of Starch by Infants.*

By J. M. Keating, M. D.

Visiting Obstetrician to the Philadelphia Hospital, and Lecturer on Diseases of Women and Children.

Recently, in a late English work, I find the following: "During the first few months farinaceous food of every kind should be avoided, for the child's stomach (?) cannot digest it. Until the third month, or even later, no saliva is secreted, and without this floury foods cannot be assimilated."† This idea is so prevalent, and most of us have adopted the statement as representing the teaching of physiologists, that it has always been a matter of surprise to those interested in the feeding of infants to find occasionally, especially amongst the poorer classes, infants fed upon corn starch or other farina, almost to the exclusion of other food, and thrive.

At present we presume that amylaceous material has of necessity to be converted by hydration into glucose, and for this reason I will not detain you this evening by indulging in the more speculative aspect of this subject, as to whether dextrine is capable of being absorbed, and which of the two ferments, that of the salivary glands or of the pancreas, is of the most importance. We have left this matter for further investigation. Prof. Albert R. Leeds, in his very able *expose* of the subject of foods as regards their chemical constituents, made the matter so clear that in his table of the analyses we have evidently an accurate guide for the selection of foods in individual cases. But as the general belief is that for infants of a tender age our choice should fall on that which contains a minimum quantity of starch, and a maximum amount of vegetable albuminoids, or foods based on the Liebig formula, I

---

* Read before the College of Physicians of Philadelphia, June 6, 1883.

† Management of Infants, etc., by Howard Barrett.

deemed it valuable to institute a series of experiments, the result of which I confess were rather surprising, to test the saliva of infants, and to satisfy myself that the reason why some children apparently thrive on starchy food is not due to any change in the starch in its preparation, but depended upon contact with secretions well established in childhood.

In these tests we endeavored, as far as possible, to exclude all error. Corn-starch was used, it having been previously boiled, cooled into a paste, and portions of this was put into little linen bags, and given to infants to suck for two minutes at a time. Pavy's test was then used; the corn-starch paste exhibited before the experiment no evidence of sugar change.

The linen was thoroughly boiled without discoloration of the solution. The bags with their contents were in each case thrown into a test-tube, and I submit for your examination the accompanying report:

| Child's Name. | Age. | No. of Teeth. | Food. | Reaction. | No. of Experiments. |
|---|---|---|---|---|---|
| Smith.... | 6 days..... | 0 | Breast .................. | None ........ | 3 |
| Coyle ..... | 7 days..... | 0 | Breast.................. | Marked...... | 1 |
| Gallagar .. | 11 days..... | 0 | Breast.................. | Well marked. | 2 |
| Asbulson.. | 12 days..... | 0 | Breast .................. | Marked...... | 1 |
| Perry ..... | 2 weeks.... | 0 | Breast .................. | ? | 1 |
| McErwin.. | 3 weeks.... | 0 | Breast and crackers...... | Well marked | 3 |
| Meenan... | 3 weeks.... | 0 | Bottle .................. | Perceptible .. | 2 |
| Conner.... | 4 weeks.... | 0 | Breast.................. | Marked...... | 2 |
| Davis..... | 4 weeks.... | 0 | Breast.................. | Marked...... | 1 |
| Beatty.... | 4 weeks.... | 0 | Breast .................. | Very slight... | 2 |
| Sumley ... | 4 weeks.... | 0 | Breast.................. | Marked ..... | 1 |
| McCann .. | 4 weeks.... | 0 | Breast.................. | Very marked. | 1 |
| Newhouse. | 7 weeks.... | 0 | Breast.................. | Very slight... | 2 |
| Roberts ... | 2 months... | 1 | Breast ................ | Marked ..... | 2 |
| Nerain .... | 2 months... | 0 | Breast.................. | Very marked. | 1 |
| Boeuning.. | 2 months... | 0 | Breast.................. | Marked...... | 1 |
| Hemileth.. | 4 months... | 2 | Corn-starch and crackers. | Well marked. | 2 |
| Roach .... | 5 months... | 1 | Corn-starch and crackers. | Slight ... ... | 3 |
| Hall...... | 8 months... | 2 | Breast and crackers...... | Marked ..... | 1 |
| Devine ... | 13 months... | 4 | Corn-starch and crackers. | Well marked. | 1 |
| Wood..... | 17 months... | | Condensed milk......... | None ....... | |

To the resident physicians of the Philadelphia Hospital, Drs. B. F. Hawley and A. E. Roussel, I am indebted for assistance in this matter, as many of these tests were repeated a number of times by them, and great care was used to insure accuracy. My report includes the results obtained by experiments with the saliva of twenty-one children, varying in age from six days to seventeen months. The sugar change was noted in all but three—one of these was a babe six days old—whilst in another babe seven days old a marked reaction was observed. I feel satisfied that some infants do digest starch provided it is presented to them in a digestible form, and also that the salivary secretion, which occurs earlier than we have been accustomed to believe, is allowed to come in contact with it, and I cannot but attribute the many statements to the effect that starchy food in small quantities is contra-indicated to the fact that the secretions of the mouth are less liable to exert their influence when such food is administered by bottle and deposited in a surprisingly short time in the acid juices of the stomach. If starchy food, such as barley-flour, oatmeal, rice, wheat-flour, etc., is indicated on account of its highly nutritious qualities which exist in all portions of the grain and deemed advisable in the feeding of children, our few observations teach us that they should be administered in such a way as to insure their thorough digestion, and I am satisfied that the surprising results we witness, especially among the poor, of thriving table-fed babes, is due to the mode of feeding more than to the fact that they are exceptional and astonishing cases. My table shows that the age of the infant is not a guide to the quality of its saliva, and we should bear this in mind when choosing the form of food. Thus, should we be called upon to regulate an infant's feeding, it would be important for us to test the saliva.

If we find a sugar change taking place we might incorporate with milk small quantities of one of the cereals, either barley, oatmeal, or wheat. On the contrary, should the test prove negative, Horlick's or Mellin's food would be decidedly preferable. But while thoroughly convinced that the saliva is a most important element in digestion we cannot overlook the fact that starchy foods have also to run the gauntlet of the pancreas, which organ, if it possess in childhood relatively the same power that it does in latter years, is far more active than the salivary glands. We cannot, then, overlook the value of a microscopic examination of the stools of all bottle-fed children, for I believe that by this alone we can regulate the quantity of farinaceous food, detecting the proportion of the undigested residue.

The following are my conclusions:

The saliva of some infants possesses the property of converting starch into glucose, regardless of age.

The age of the infants cannot be taken as an indication of this property of its saliva.

When such a condition is found to exist a small quantity of well-prepared farinaceous food is valuable as an element in the diet, incorporated with mixed cow's milk.

An examination of the stools of children so fed would be a guide as to the quantity of starchy food to be used, and when farinaceous food is employed slow feeding is probably preferable to the bottle.—*The Boston Medical and Surgical Journal.*

